# Human data at odds and in confirmation of the two-process model of sleep regulation—a perspective

**DOI:** 10.1038/s44323-026-00083-3

**Published:** 2026-06-22

**Authors:** Derk-Jan Dijk

**Affiliations:** Surrey Sleep Research Centre, Faculty of Health and Medical Sciences, University of Surrey, Guildford, UK

**Keywords:** Circadian rhythms and sleep, Diseases

## Abstract

This perspective summarises human data collected in experiments conducted over a 40-year period designed to test the two-process model of sleep regulation in which sleep is regulated through the interaction of circadian rhythmicity and sleep homoeostasis. The perspective highlights findings in accordance with and at odds with predictions and assumptions of the model. Implications for understanding how circadian and sleep-wake dependent processes interact to regulate homoeostasis of brain function are discussed.

## Preamble and scope

Now, more than 40 years ago, the author of this perspective was recruited to a PhD studentship (1983–1988) at the University of Groningen, the Netherlands. The PhD studentship was about testing the predictions of the two-process model of sleep regulation, and this was to be done in humans and animals, but in the end, most of the thesis was about humans^1^. The local multidisciplinary team of PhD supervisors comprised a physicist (Domien Beersma), a biologist (Serge Daan), and a psychiatrist (Rutger Van Den Hoofdakker). My initial reading list consisted of (1) a paper published in Human Neurobiology- a journal which no longer exists- with the title: A two process model of sleep regulation and (2) a manuscript with the title ‘Timing of human sleep: recovery process gated by a circadian pacemaker. If that wasn’t enough immersion into circadian and sleep-homoeostasis thinking and exposure to multi-disciplinarity, there were regular face-to-face meetings between the Groningen scientists and Alexander A Borbély (a MD-neuroscientist), Irene Tobler (a zoologist) and other sleep researchers in the Institute of Pharmacology at the University of Zurich. During these meetings, we reviewed human and animal data and planned experiments to test the two-process model.

The two papers on my reading list^2,3^, which I will refer to as 2PM-1982 and 2PM-1984, are now recognised as seminal. They present a conceptual and quantitative modelling account of the regulation of sleep by the interaction of a relaxation oscillator driven by the alternation of sleep and wakefulness (Process S) and a self-sustained circadian oscillator (Process C) not affected by the sleep-wake cycle. Currently, this concept is most often summarised as ‘sleep is regulated by sleep homoeostasis and circadian rhythmicity’.

The two seminal papers and associated concepts have guided other researchers and me, and continue to be cited frequently and by many. The history and contributions of the 2PM have been reviewed repeatedly^4–9^. Here, I present my assessment of the status of the 2PM and the concept of sleep regulation by circadian rhythmicity and sleep homoeostasis. The perspective is mostly based on my research in humans conducted during my stays at the University of Groningen, the University of Zurich, Harvard Medical School, the close collaboration with Pierre Maquet’s group at the University of Liege and the work conducted in the Surrey Sleep Research Centre at the University of Surrey. A review primarily focusing on animal experiments and the 2PM has been published recently^10^. The mathematical structure of the 2PM and its relation to other coupled oscillator models in physiology have been presented in this journal^11^. The current perspective highlights empirical findings in humans that are in accordance with the assumptions and predictions of the 2PM and those that are at odds with the 2PM. The perspective is inspired by the belief that we either ignore models or take the assumptions and quantitative aspects of models seriously.

The 2PM-1984 assumption that light enters the entrainment pathway primarily through the circadian pacemaker is re-evaluated and clarified. A central claim of the 2PM is that EEG slow-wave activity (SWA), i.e. EEG power density in the 0.75–4.5 Hz range during NREM sleep, is a marker of Process S, sleep homoeostasis, sleep intensity, and the recovery function of sleep. Assumptions about the physiological correlates and quantitative aspects of the S process, i.e. its association with SWA, the time constants of the exponential processes and their putative association with brain recovery processes, are critically discussed. The concepts of sleep intensity and sleep homoeostasis are revisited, and a rethink of the meaning of these concepts is encouraged. The assumption in both the 2PM-1982 and 2PM-1984 models that in humans the circadian drive for wakefulness is highest in the afternoon is shown to be incorrect: it is highest in the evening. Analyses of several aspects of sleep and brain function demonstrate that, unlike originally postulated, processes S and C interact continuously and in a more complex manner than a simple addition. Overall, the data show the value of the core concepts of the 2PM, but also that not all the assumptions and concepts have survived the test of time and data. The confirmation and refutations of some of the quantitative and conceptual aspects of the 2PM are made explicit here, not only because they are relevant for basic research but also because they have implications for understanding the regulation of sleep and waking function of humans living in the real world.

The findings and their implications are summarised in a conceptual framework in which homoeostasis is maintained through the interactions of circadian and sleep-dependent processes.

## Light and the two-process model of sleep regulation: anatomy vs systems approaches

### Light reaches the circadian process via the sleep-wake cycle

**Fig. 1:**
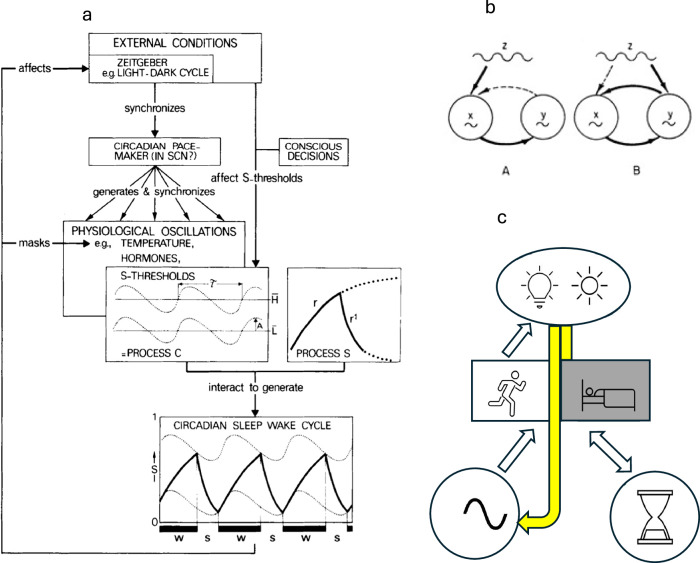
The two-process model of sleep regulation and how light enters the human timing system. **a** Schematic representation of the two process model^3^. Please note the arrow from the sleep-wake cycle to the zeitgeber box, indicating how the sleep-wake cycle may affect the zeitgeber. **b** Schematic description of Kronauer’s view of the difference between the 2PM and the X-Y model such that in the 2PM the zeitgeber Z, directly affects the circadian pacemaker (X), whereas in Kronauer’s X-Y model the effects of Light enter the system through the Sleep-Wake Oscillator (Y)^109^. **c** Schematic representation of how light (both human-made and natural light) reaches the circadian pacemaker through the sleep-wake cycle (i.e. we keep the human-made light on when we are awake and light can reach the pacemaker; turn off the light/close our curtains and eyes when we go to sleep, and this prevents light from reaching the circadian pacemaker). The sleep-wake cycle is driven by the combined influence of the circadian pacemaker (Process C) and the hourglass oscillator (Process S). The bidirectional arrow between the sleep-wake cycle and the hourglass oscillator indicates that the hourglass is both driving the sleep-wake cycle and is driven by the sleep-wake cycle.

Given that the endogenous near 24-h oscillator involved in sleep timing needs to be synchronised to the 24-h light-dark cycle, understanding how light impacts this system is of relevance for our understanding of sleep timing in society. This is evident from the claims and counterclaims about entrainment to sun time^12^, continuing discussions about the merits and perils of daylight saving time^13,14^, and discussions about causes of social jet lag and delayed sleep-wake phase disorder^15^. In the 2PM-1984, it was asserted that light directly influences the circadian oscillator, even though in the schematic representation it was indicated that sleep may affect the ‘zeitgeber’ (Fig. 1a). Such a direct influence of light on the circadian pacemaker was in accordance with general circadian entrainment theory, which was based on research in animals with a polyphasic sleep pattern. It was also in accordance with anatomy: the retino-hypothalamic tract (RHT) directly connects the retina to the SCN. This ‘direct light effect on the circadian oscillator’ was at odds with the two-oscillator (X and Y) model of Richard Kronauer and colleagues^16^. In fact, in a commentary attached to the 2PM-1984 paper, Kronauer explicitly criticised the 2PM view that light directly affects the circadian pacemaker (Fig. 1b). Based on his analyses of the change in phase relationship between the monophasic sleep-wake (Y) and temperature oscillator (X) upon release from entrainment into free run, and a phenomenon called phase trapping, Kronauer postulated that light enters the sleep-wake-circadian-timing-system through the sleep-wake oscillator. This model, which was very much based on an analysis of phenotypical characteristics of the human circadian timing system, rather than anatomy, predicted that light exposure would not shift circadian rhythms if the sleep-wake cycle is kept constant during the light exposure. This intensely discussed controversy was addressed in experiments conducted by me and collaborators in Groningen^17,18^ and in a case study conducted by Charles Czeisler and his team at Harvard Medical School^19^. The data demonstrated that the melatonin, core body temperature, cortisol and sleep propensity rhythm could be shifted by light even when the sleep-wake cycle was kept constant.

These data favoured the ‘direct light access to SCN ‘hypothesis. But were Kronauer and colleagues really wrong and Daan, Beersma and Borbely right? In the real life of adult humans, light enters the system through the monophasic sleep-wake cycle: we keep the lights on until we go to sleep and keep the lights out when we sleep. In other words, light input to the SCN is gated and, for all practical purposes, driven by the sleep-wake cycle. This light pathway is presented in Fig. 1c, in which for light to reach the circadian oscillator, it goes through the sleep-wake cycle. The sleep-wake cycle, in turn, is driven by the combined influence of the circadian process and the hourglass process (Process S). This latter process is driven by the sleep-wake cycle. Early mathematical modelling of this hierarchical entrainment structure showed that a systems approach can indeed explain phase trapping^20^. Recent models incorporating this notion of gating of light input, combined with knowledge about the phase-dependent response and the sensitivity of the pacemaker to light, show how the light-dark exposure generated by the sleep-wake cycle delays our clocks and causes social jet-lag^12,21,22^. These extensions of the 2PM in which the timing of sleep is modelled as a hierarchical entrainment system with feedback through the sleep-wake cycle are powerful tools aiding our understanding of sleep timing. They provide a comprehensive context to discussions about school start times, working schedules, the importance of sun-time vs social time, the interpretation of social jet lag, and other sleep phenotypes, as well as the design of interventions^23,24^.

### Light effects beyond circadian phase shifting

It is now well established that light regulates circadian entrainment by interacting with the core molecular mechanisms generating circadian rhythmicity^25^. These effects of light are mediated by melanopsin-expressing retinal ganglion cells and their projections to the SCN. The ganglion cells also project to other parts of the brain, and light can thereby exert effects on brain function that extend well beyond phase shifting and entrainment of circadian rhythmicity. These effects include modulation of alertness, mood and cognition^26^. Effects of light on brain function during wakefulness, as assessed by fMRI, have been shown to depend on the status of the ‘sleep homeostat’ and circadian phase^27^. Early animal experiments had documented the direct effects of light and darkness on sleep^28^. More recently, evidence has emerged that light affects parameters of the sleep-wake oscillator. Thus, light has been reported to increase the rate at which wakefulness leads to the buildup of sleep pressure as indexed by SWA, which, according to the 2PM, is a marker of sleep homoeostasis^29,30^.

Overall, human data collected since the publication of the 2PM convincingly show the intricate interactions of light, circadian rhythmicity, and the sleep-wake dependent process. To some extent, these data are at odds with the 2PM at which these processes were seen as independent. It is, however, to the credit of the 2PM that it recognised the relevance of light in the interaction of circadian rhythmicity and sleep, which led to testable hypotheses, new insights and new integrative mathematical models.

## Phenomena at odds with slow wave activity as a biomarker of recovery and sleep homoeostasis

### Parameters describing the recovery process and sleep homoeostasis

How and how fast sleep recovers brain function after a period of wakefulness is relevant for our evaluation of sleep-wake patterns in humans. In the 2PM, slow-wave activity, defined as EEG power density in the 0.75–4.5 Hz range during NREM sleep, was stated to reflect Process S, a marker of sleep homoeostasis and an index of sleep intensity and the recovery process (2PM-1982,2PM-1984). In 21st-century terminology, SWA was considered a biomarker of Process S. But what are the quantitative characteristics of SWA across sleep-wake manipulations and does it indeed track brain recovery during sleep? As a biomarker of process S, SWA should increase in a saturating exponential manner in response to the duration of wakefulness preceding sleep and decrease exponentially during sleep. The exponential nature of the S process, rather than, for example, a linear increase and decrease, has important implications. It implies that each successive hour of wakefulness contributes a smaller increment to sleep pressure and each hour of sleep a smaller decrement in sleep pressure. In other words, according to the 2PM-1982, 2PM-1984, sleep has an intensity dimension [see also ref. ^6^].

**Fig. 2:**
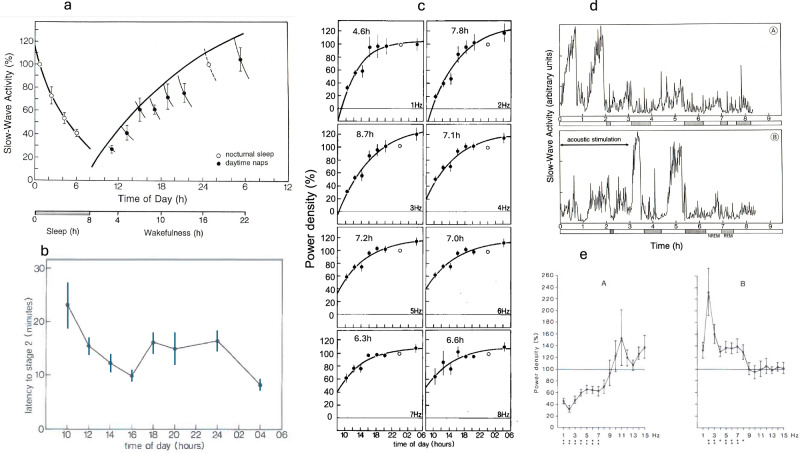
SWA, EEG power density and sleep propensity during nocturnal sleep, daytime naps and during and following SWS suppression. **a** SWA during nocturnal sleep and Daytime naps. SWA was averaged per NREM period during nocturnal sleep (open symbols), and naps initiated at 10, 12, 14, 16, 18, 20 and 04 h (closed symbols). Averages and SEs are plotted at the midpoint of NREM periods. The level of S at sleep onset was estimated by applying the standard decay function of SWA over the duration of the NREM periods (which varied across naps from approximately 60 min at 10 am to 122 min at 04 am and is proportional to the thin lines). The continuous bold lines represent the time course of Process S during nocturnal sleep and during wakefulness using the time constants of ref. ^3^. Note that naps were scheduled on different days to avoid the impact of one nap on subsequent naps^20^. Modified from ref. ^110^. **b** Sleep latency during daytime naps and sleep initiated at midnight and 4am. Note midafternoon dip and dissociation between the diurnal profile of sleep propensity (**b**) and SWA (**a**)^32^. **c** EEG power density during the first 30 min of daytime naps and nocturnal sleep: dynamics vary across frequencies. All values are expressed as % of the corresponding values during the first 30 min of baseline sleep. Note the rapid increase (short time constants) of low frequency activity, and the slower increase of theta activity with time awake. Modified from ref. ^32^. 1 Hz = power density 0.25–1.0 Hz, 2 Hz = 1.25–2.0 Hz, etc. Numbers at the top of panels are time constants in hours of an experimental saturating function fitted to the data. **d** SWA during an undisturbed night and a night during which SWA was suppressed by auditory stimulation. During the first three hours of the second night (lower panel), acoustic stimuli were delivered to the participant contingent upon the appearance of slow waves. By adjusting the intensity of the stimuli, slow wave activity could be suppressed without inducing wakefulness. After the 3 h stimulation period, SWA rebounds, i.e. values were higher than during the corresponding time interval during the undisturbed night (upper panel). Modified from ref. ^110^. **e** Changes in the EEG power spectrum in NREM sleep during (A) and after (B) SWS suppression. Values (averages and SEs) are expressed relative to the corresponding values during the undisturbed night. Note that during the SWS suppression and during the rebound, largest changes are observed in the 1.25–2.0 Hz bin and that significant effects (indicated by asterisks) extend up to 7 and 8 Hz, as was also observed in the data presented in (**c**)^54^.

**Fig. 3:**
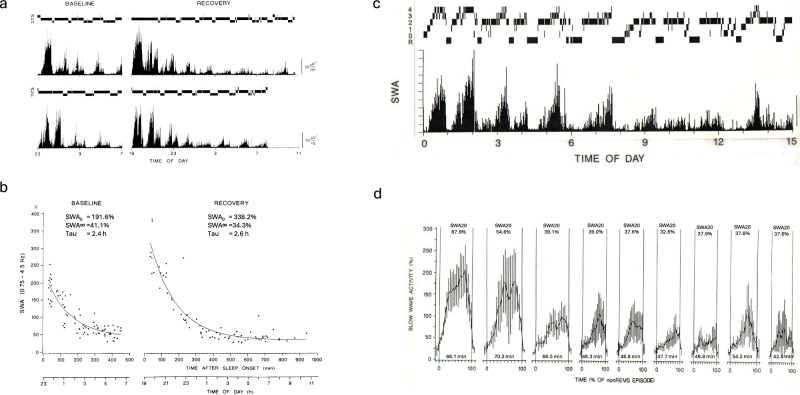
Dynamics of SWA during long sleep. **a** Examples of SWA during baseline and during extended recovery sleep, initiated at 19:00 h after 36 h of sleep wakefulness. Reduced hypnogram at top of panels *W* = Wakefulness, *N* = N1, N2, SWS combined; *R* = REM sleep. **b** SWA during baseline and recovery sleep following 36 hours of sleep deprivation. Individual data points represent SWA averaged per NREM episodes. Data are expressed as a percentage of SWA averaged over the baseline sleep period. Note the higher initial values in recovery sleep compared to baseline and the leveling off of the decay of SWA. When fitting the function SWAt = SWAo X e^-t/tau^ + SWA∞ (Solid line) to the data, the initial values for SWA were 191.6 and 338.2, the asymptotic values for SWA were 41.1 and 34.3%, and the time constants were 2.4 and 2.6 h for baseline and recovery sleep, respectively. Modified from ref. ^38^. **c** Example of the time course of sleep stages and SWA during extended sleep initiated at midnight. Sleep stages were scored according to Rechtschaffen and Kales' criteria. Note reoccurrence of stages 3 and 4, i.e. SWS, between 13:00 and 14:00 h. SWA during this period is somewhat higher than in the preceding hours, but much lower than during SWS at the beginning of the sleep period (Dijk et al.). **d** Dynamics of SWA during the first 9 NREM episodes of extended sleep initiated at midnight. To illustrate the dynamics of SWA, each NREM period was subdivided into 20 bins (5% each) and average SWA was calculated across participants for each of these bins. Average SWA in the first 20 min of each NREM period is indicated at the top. The width of each NREM episode is proportional to the average duration of these NREM episodes, which is indicated at the bottom of each panel. At the beginning of the sleep period, NREM episodes are longer than in the second part of the sleep period. All values are expressed as a percentage of the average SWA in NREM sleep. Modified from ref. ^31^.

One question is how to estimate Process S from SWA data. SWA displays ultradian dynamics (Figs. 2d and 3d), i.e. within a NREM episode it rises from the beginning until the end of a NREM period and then falls abruptly to low levels just before REM sleep. Thus, SWA not only depends on time awake and time asleep but also on the NREM-REM cycle, and it correlates with the duration of NREM periods^31^. It follows that the saturating exponential increase of SWA with time awake, as predicted by the 2PM, is only observed when SWA is averaged over an NREM period. In fact, the most accurate reflection of Process S in SWA is obtained when SWA is averaged over a fixed duration of NREM sleep.

Nocturnal sleep and daytime nap studies, conducted as part of my PhD project in Groningen, demonstrated that within a physiological range of wake duration of 2, 4, 6, 8, 10, 12, 16 and 20 h, low frequency EEG activity in the first NREM sleep episode indeed increased monotonically with time awake. This increase could be fitted with an exponentially saturating function^20,32^, provided that the variation in the duration of the NREM episode was taken into account (Fig. 2a). This monotonic increase contrasts with the diurnal pattern of sleep propensity as assessed by latency to sleep onset, which is in accordance with the notion that sleep propensity not only depends on Process S but also time-of-day, i.e. circadian rhythmicity. (Fig. 2b). A limitation of these nap studies is that no naps were scheduled after 14 h of wakefulness, which would coincide with what is now known as the wake maintenance zone (see below).

Even though activity in the 0.25–8 Hz range increased monotonically with time awake, the time constants, i.e. the rate at which time awake affected EEG oscillations or to be more precise the time it takes to reach 63.2 % of its asymptotic value, is very much dependent on the EEG frequency considered (Fig. 2c). The time constant for EEG activity in the 0.25–1 Hz range averaged over the first 30 min of NREM sleep, is as short as 4.6 h (compared to 18.2 h for Process S in 2PM-1984), which implies that after 16 h of wakefulness this variable is at approximately 85% of its asymptotic value. Delta and theta frequencies displayed a slower increase with time constants ranging from 8.7 to 6.3 h. Although this variation in the time constants is not at odds with the 2PM it illustrates the intricacies of estimating parameters of the model and begs the question which time constant represents Process S.

The exponential decay of process S can also only be observed when SWA is averaged across an NREM period. In fact, the time constant of the decrease of process S in 2PM-1984 was based on an analysis of EEG power density in the first three NREM-REM cycles (i.e. the average included both NREM and REM) of a 7.5 h nocturnal baseline sleep period and a recovery sleep period after 40.5 h of wakefulness^33^. The time constant for the decay of process S during sleep, i.e. the time it takes to reach 36.8% of its asymptotic value, was estimated to be 4.2 h. However, already in this early study, it was noticed that both the decay of EEG activity during NREM sleep and the response to sleep deprivation vary across EEG frequencies in the 0.5 to 4.5 Hz range. The very slow oscillations (<1 Hz) display not much of a decay during sleep and not much of an increase with sleep deprivation. In contrast, EEG activity in the delta frequencies is very sensitive to variation in sleep-wake duration. This differential response has been observed in a range of experimental paradigms, one of which is selective SWA deprivation. When SWA is suppressed during the first part of the nocturnal sleep period by acoustic stimulation, a rebound is observed in the second part of the sleep period (Fig. 2d). Both the direct response to acoustic stimulation (Fig. 2e_A) and the subsequent rebound (Fig. 2e_B) depend on the EEG frequency considered, with activity in the 1–2 Hz range always showing the largest response. Although not controversial, this heterogeneity of low-frequency EEG oscillations in response to sleep-wake manipulations is relevant, for example, in the context of the significance of the slow-oscillation and delta waves for recovery of brain function^34^ and is gaining more and well-deserved attention. One striking example of the differences between low-frequency activity (slow oscillations) and delta activity relates to the relevance of slow EEG activity in NREM sleep for Alzheimer’s disease^35^. EEG activity in the 0.6–1 Hz range was correlated negatively with PET-assessed amyloid burden, whereas the opposite association was reported for EEG activity in the 1–4 Hz range.

The estimate of the time constants of the recovery process not only depends on the frequency range considered. Over the years, reported estimates of the time constants of SWA have varied considerably, with the original value of 4.2 h being revised to 2.2 h or shorter in some cases^36^. Some of this variation may be indicative of individual differences in ‘process S’.

Valid estimates of the time constants of the sleep-wake dependent cycle of the deterioration of brain function during wakefulness and its recovery during sleep are not only of theoretical interest. If we assume that SWA reflects a brain recovery process, the time constants of this process have a direct bearing on how we interpret the importance of the loss of 1,2, 3 or 4 h of sleep. Shorter time constants of the recovery process imply a faster recovery and hence a smaller negative impact of the loss of the final hours of sleep. This is particularly relevant for our understanding of the negative effects of moderate sleep restriction common in society, e.g. 6 h of sleep rather than 7.5 h, on brain function (see below).

### Reoccurrence of slow wave activity at the end of long sleep episodes

An early controversy in the context of SWA as a biomarker of an hourglass oscillator was related to the question of whether the decay of SWA during sleep continues indefinitely. Broughton and colleagues had reported a recurrence of SWS, i.e. visually scored stages 3 and 4 of NREM sleep according to the Rechtschaffen and Kales criteria, after approximately 12 h of sleep and postulated a 12-h oscillator underlying SWS^37^. Clearly, this was at odds with SWA being a biomarker for Process S, at least when we assume that visually scored SWS and SWA derived from the Fourier transform are closely related measures of slow-wave activity. To address this issue, I designed and conducted two experiments while a post-doc in Borbely’s lab^31,38^. The design of the first experiment was based on the observation in classical free-running experiments that sleep periods initiated in the early circadian evening, i.e. approximately 10–15 h before the temperature minimum, were very long, up to 12–16 h^39^. Indeed, when our young participants were kept awake for 36 h and went to sleep at 19:00 h they slept for 12–16 h, generating 7–8 NREM-REM cycles (Fig. 3a, b). In a second experiment, the young participants were not sleep deprived and went to bed at midnight and were instructed to sleep as long as possible. And long they slept, sometimes for as long as 15 h, producing many NREM-REM cycles (Fig. 3c). In both experiments, not much of a recurrence of periods of SWA was observed, although an occasional moderate peak in SWA occurred (Fig. 3a–d). The occasional peaks of SWA at the end of the sleep period easily lead to scoring of SWS. The visual scoring is based on a threshold criterion: SWS with little SWA can by visually scoring not be distinguished from SWS with much higher SWA at the beginning of sleep. In other words, visual scoring led one to believe that there was a substantial recurrence of SWA, whereas quantitative analyses showed this recurrence to be minor (Fig. 3c, d). Nevertheless, the occurrence of any SWA increases at the end of long was to some extent at odds with the 2PM and required an explanation. The recurrence of SWA at the end of these long sleep periods was reconciled with Process S by taking into account the variation in the duration of NREM periods and, importantly, by assuming that S increases also during REM sleep. Because REM sleep is under circadian control, the very long REM periods at the end of sleep lead to an increase in S and SWA in subsequent NREM periods, provided these are of sufficient duration^40^.

### The high asymptotic values of the slow wave activity decay function during sleep

During these long sleep periods^31,41^ another characteristic of the time course of SWA emerged, which may be considered problematic within the context of SWA as a biomarker of the recovery process. The very long sleep periods with many NREM periods allowed for a robust quantitative estimation of the time course of SWA. The fitted function I applied not only estimated the time constant of the decay but also the asymptote, i.e. the level of SWA if sleep were to continue indefinitely. The fitting demonstrated that the asymptote of the decay was not zero but rather high, at approximately 35–41% of the average SWA during baseline sleep. When this asymptote was included in the fitted function, the time constant of the exponential decay turned out to be rather short (2.4–2.6 h, vs. 4.2 h in 2PM1984) and (Fig. 3b, d)^38^. In other words, the effects of prolonged wakefulness on SWA dissipate within a few hours of sleep, and thereafter, no further decrease of SWA is observed. This poses somewhat of a conundrum within the framework of SWA as a marker of a recovery process. If, for example, sleep is for downscaling of synapses and SWA reflects synaptic strength^42^, what is happening during all those hours of sleep during which there is no further decay of SWA? One interpretation is that a steady state has been reached, and no further recovery has taken place, but this seems rather unlikely. We postulated sophisticated mechanisms to reconcile these observations with the notion that SWA reflected process S and associated recovery of brain function^38^, but these explanations remain somewhat unsatisfactory. In rodents, the time course of SWA during recovery from sleep deprivation is also seemingly at odds with the notion that SWA is a primary indicator of the recovery process. During recovery from sleep deprivation, total sleep time remains above baseline, while at the same time, SWA, after an initial increase, drops below baseline. These anomalous ‘SWA’ observations and other observations suggest that the recovery from sleep deprivation outlasts the enhancement of SWA^10^.

### The marginal increase in slow wave activity during longitudinal sleep restriction

**Fig. 4:**
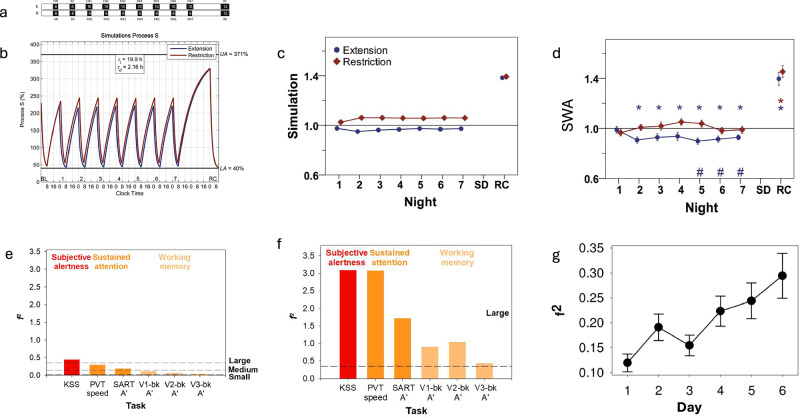
SWA and daytime function in response to sleep restriction. **a** Design of the experiment. After a Habituation and Baseline night, participants were scheduled to sleep restriction (6 h time in bed) or sufficient sleep (i.e. the sleep opportunity was extended to 10 h time in bed) for 7 nights. This was followed by a total sleep deprivation and recovery sleep. **b** Simulation of the time course of Process S during sleep and wakefulness. Note the very small increase in Process S in response to the nights of sleep restriction and the much larger response to total sleep deprivation. **c** Process S averaged across the longest common sleep duration in the two conditions. **d** Average SWA (Central derivation) during nights of sleep restriction (red) and sleep extension (blue) expressed relative to baseline (=1). # significant difference between simulations and data; * significant differences for sleep extension from baseline. No significant difference from baseline was observed for sleep restriction, but total sleep deprivation following both sleep restriction and sleep extension induced a large and significant response. (Fig. 4a–d: modified from ref. ^45^. **e** Effect of sleep restriction on Karolinska Sleepiness Scale, Psychomotor Vigilance Tasks, Sustained Attention to Respond Task, Verbal 1, Verbal 2, and Verbal 3 N-back during the last two days of sleep restriction. **f** Effect of total sleep deprivation on the Karolinska Sleepiness Scale, Psychomotor Vigilance Tasks, Sustained Attention to Respond Task, Verbal 1, Verbal 2, and Verbal 3 N-back during the day. **g** Progressive increase in the effect of sleep restriction averaged across a wide variety of daytime function measures. *f*^2^ = Effect size. Figure 4e–g modified from ref. ^48^.

Parameters of the S process in 2PM-1984 were based on baseline sleep and the increase in SWA in response to 40.5 h of acute total sleep deprivation. In real life, sleep is often short for several consecutive nights rather than completely absent for one night. Repeated partial sleep deprivation experiments have been conducted and analysed in the context of 2PM in both Zurich and Surrey and elsewhere. In this paradigm (Fig. 4a), SWA behaves in accordance with exponential functions (Fig. 4b, c): SWA shows a moderate to small increase and reaches a near steady level after only a few days of sleep restriction. When sleep is restricted to 4 h for 2 or 4 nights, SWA increases to approximately 15–20% above baseline^43,44^. After sleep restriction to 6 h for seven nights, the increase in SWA is only 6%^45^(Fig. 4d). When in the same protocol, participants undergo a total sleep deprivation of approximately 40 h, an increase to approximately 140% during recovery sleep is observed, which is close to the predicted response (Fig. 4d, RC). These empirical data are all very close to the theoretical predictions (Fig. 4b, c). Assessment of daytime function through an extensive test battery revealed that both sleep restriction and total sleep deprivation lead to deterioration, and in particular, in alertness and attention, but less so for working memory, with the negative effects of total sleep deprivation being larger (Fig. 4e, f). The main dissociation between the response of performance and SWA relates to the time course during sleep restriction. Whereas SWA reaches a steady level after only a few nights of sleep restriction, brain function during wakefulness, averaged across many tests of waking performance, continues to deteriorate (Fig. 4c, d, vs. Fig. 4g)^46–48^. Furthermore, after repeated partial sleep deprivation, SWA returns to baseline after one night of recovery sleep, but some brain functions may take several days to recover^49^. In other words, even though the response of SWA closely follows the predictions based on process S, its time course doesn’t parallel the deterioration and recovery of brain function as observed in sleep restriction paradigms. The implication is that aspects of sleep which are lost during partial sleep deprivation, such as total sleep time or REM sleep, may be as, or even more, important for the recovery process than SWA.

### Selective suppression of slow wave activity: effects and lack there of

The functional significance of SWA in the context of sleep timing and recovery of brain function in humans was investigated further using SWS deprivation paradigms, in both Groningen and Surrey and elsewhere^50–53^. In these experiments, auditory stimuli were delivered when slow waves appeared in the EEG. The acoustic stimulation of appropriate intensity leads to a suppression of SWA without awakening participants and without a change in NREM duration. This paradigm can thereby separate the contribution of NREM duration from the contribution of SWA to recovery. SWA suppression in the initial part of nighttime and daytime sleep leads to a rebound of SWA within the same sleep period (Fig. 2d), providing evidence for intranight ‘homoeostatic’ regulation, which is not regulated by NREM time but by accumulated SWA^50,54^. Slowing down the decrease of SWA by acoustic stimulation did not delay waking up from either nocturnal or daytime sleep. This result is obviously at odds with SWA being a biomarker of process S regulating sleep timing. In fact, I am not aware of any experiment in which a causal role for SWA in sleep timing has been demonstrated in humans.

However, in support of a role for SWA in the regulation of sleep propensity rather than actual timing, suppression of SWA throughout the entire sleep period increases the propensity to initiate sleep the next day, as assessed by the Karolinska Sleepiness scale and the Multiple Sleep Latency Test, to a considerable extent^52^. In further support of a role for SWA for the recovery of brain function, SWA suppression also leads to less positive affect, slower or impaired information processing and sustained attention, less precise motor control, and erroneous implementation, rather than inhibition, of well-practised actions. The size of these effects was, however, smaller than the effects on sleep propensity^51^.

Overall, the data show that, in accordance with the 2 PM, SWA is driven by the sleep-wake cycle. The data also show that the short time constant of the time course of SWA during sleep does not reflect the time course of ‘brain’ recovery. Thus, the dissociation between SWA and recovery of brain function is at odds with the assumption in the 2PM that SWA is an accurate marker of sleep intensity and a marker of the variables that are homeostatically regulated by sleep. The dissociation implies that the time constants of the recovery processes served by sleep vary across dependent variables.

### Relative and absolute measures of slow wave activity and whether they relate to sleep regulation

Although earlier studies, in particular those by Wilse Webb and Irwin Feinberg, had described many aspects of the regulation of slow wave sleep and slow waves, a strength of the 2PM is the explicit and quantitative link between process S and a quantitative physiological marker assessed by appropriate signal analysis methodology, i.e. SWA during NREM sleep. To this day, different approaches to quantify the homeostatic aspect of sleep are used. Whether ‘intensity’ measures, such as SWA or duration measures, such as time in NREM sleep or time in SWS, or sleep continuity measures, are most relevant from a brain recovery perspective has been discussed repeatedly for human sleep^55,56^, and the answer remains unclear.

**Fig. 5:**
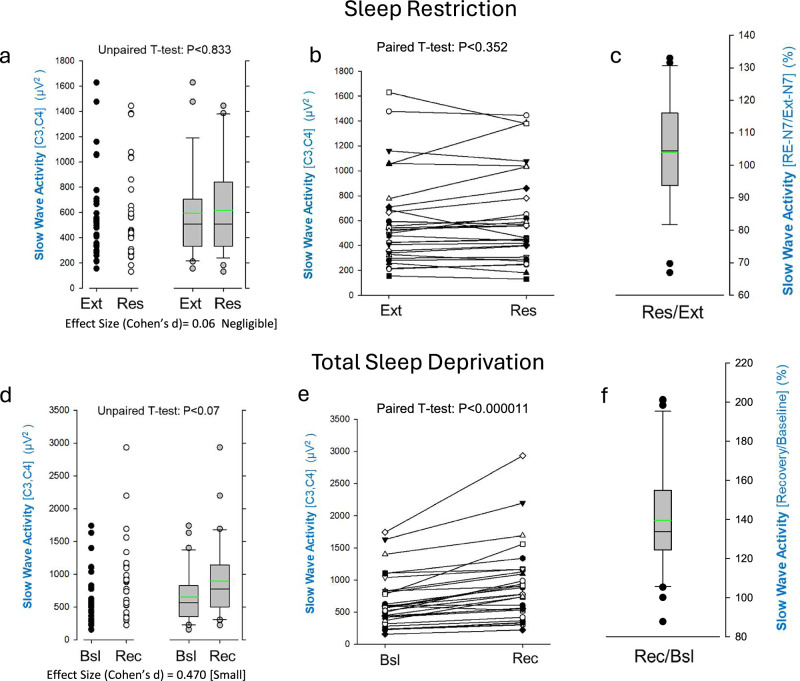
Individual differences in the absolute values of SWA and response to total and repeated partial sleep deprivation. **a** Slow wave activity (EEG power 0.75–4.5 Hz, in microV^2^) during the 7th night of sleep extension (Ext, 10 hour sleep opportunity) or Restriction (Res, 6 hour sleep opportunity). Box plots illustrate quartiles, medians and means. The effect size of sleep restriction vs. sleep extension when computed on the absolute values is negligible. **b** Same data as in (**a**), but now data belonging to the same participants are connected, allowing a within-individual comparison and demonstrating the very small and inconsistent response to sleep restriction. **c** When, for each individual, the ratio of SWA during Restriction over Extension is computed for the common sleep duration, SWA is approximately 106% (100% = extension). **d** Slow wave activity during Baseline (Bsl) and Recovery (Rec) sleep in healthy young adults. Each data point represents the average SWA of one participant during baseline sleep and sleep following sleep deprivation. Box plots illustrate quartiles, medians and means during baseline and recovery sleep. The effect size of sleep deprivation, when computed on the absolute values, is small. **e** Same data as in (**d**), but now data belonging to the same participants are connected, allowing a within-individual comparison and demonstrating the consistent response to total sleep deprivation. **f** When, for each individual, the ratio of SWA during Recovery over Baseline is computed for the common sleep duration, SWA is approximately 140% (100% = baseline). Previously unpublished figure based on data published in ref. ^45^.

Even if we accept SWA as a useful marker of the homeostatic sleep process, there are questions about how it should be measured or presented. In most 2PM studies, SWA is expressed as a percentage of baseline. Absolute values which vary substantially across individuals, age groups and sexes, are rarely considered. In humans interindividual differences in SWA are so large that they exceed the effect size of both sleep restriction (Fig. 5a) and total sleep deprivation (Fig. 5d). In fact, when applied to absolute measures in a cross-sectional approach the effect size of sleep restriction is negligible (Fig. 5a) and the effect of total sleep deprivation is small (Fig. 5d). Effects of several days of sleep restriction remain very small even in a within comparison (Fig. 5b, c). The effect of total sleep deprivation becomes only apparent in a within-participant comparison, which always yields a SWA value of approximately 140% during recovery sleep when the baseline is set at 100% (Fig. 5e, f).

The interpretation of differences in absolute values of SWA as observed across the sexes and age groups, within the context of the 2PM remains unclear^52,57,58^. For example, it is well established that compared to men, women have higher levels of SWA^59,60^. At first sight, this may indicate a sex difference in ‘sleep homoeostasis’, which would fit well with Rutger Wever’s report that in an environment free of time cues, women slept much longer than men^61^. However, comparing the EEG spectra profile of the sex differences demonstrated these sex differences to be very dissimilar from the effects of sleep deprivation^59^. This argues against the interpretation that the sex related differences in SWA reflect differences in Process S. Furthermore, the sex difference in deterioration of waking performance during desynchrony of the sleep-wake cycle of circadian rhythmicity appears not to be related to the sleep-wake dependent process but rather to a larger circadian amplitude in women^57^. Processes associated with differences in the absolute value of SWA may be different from those involved in the homeostatic response. Thus, interindividual differences in SWA are to some extent related to structural differences such as myelination, whereas the response to sleep loss is not related to these structural aspects^62^.

Another issue relates to the protocols used to quantify sleep homoeostasis as indexed by SWA. ‘Sleep homoeostasis’ is most often quantified by the change of SWA from baseline after a single dose of wakefulness. This is somewhat problematic because in any non-linear process, such as Process S, an increase after a dose of wakefulness not only depends on the dose but also on baseline values. To be precise, given a fixed time constant of the increase of process S, a single wake duration challenge will lead to a much larger increase when the initial values are lower. Quantifying the parameters of the S process of the 2PM requires proper dose-response experiments, but few have been conducted.

The reliance on quantifying sleep homoeostasis by measuring changes in SWA in many experiments conducted within the context of the 2PM has left the interpretation of the absolute values of SWA underexplored. On the other hand, in many experiments it is tacitly assumed that differences in absolute values of SWA reflect differences in ‘sleep homoeostasis’.

### Circadian modulation of slow wave activity

In the context of SWA as a marker of process S and the recovery function of sleep, investigating the extent to which it is modulated by circadian factors is relevant. After all, in the 2PM sleep homoeostasis and circadian rhythmicity were considered functionally and physiologically separate.

**Fig. 6:**
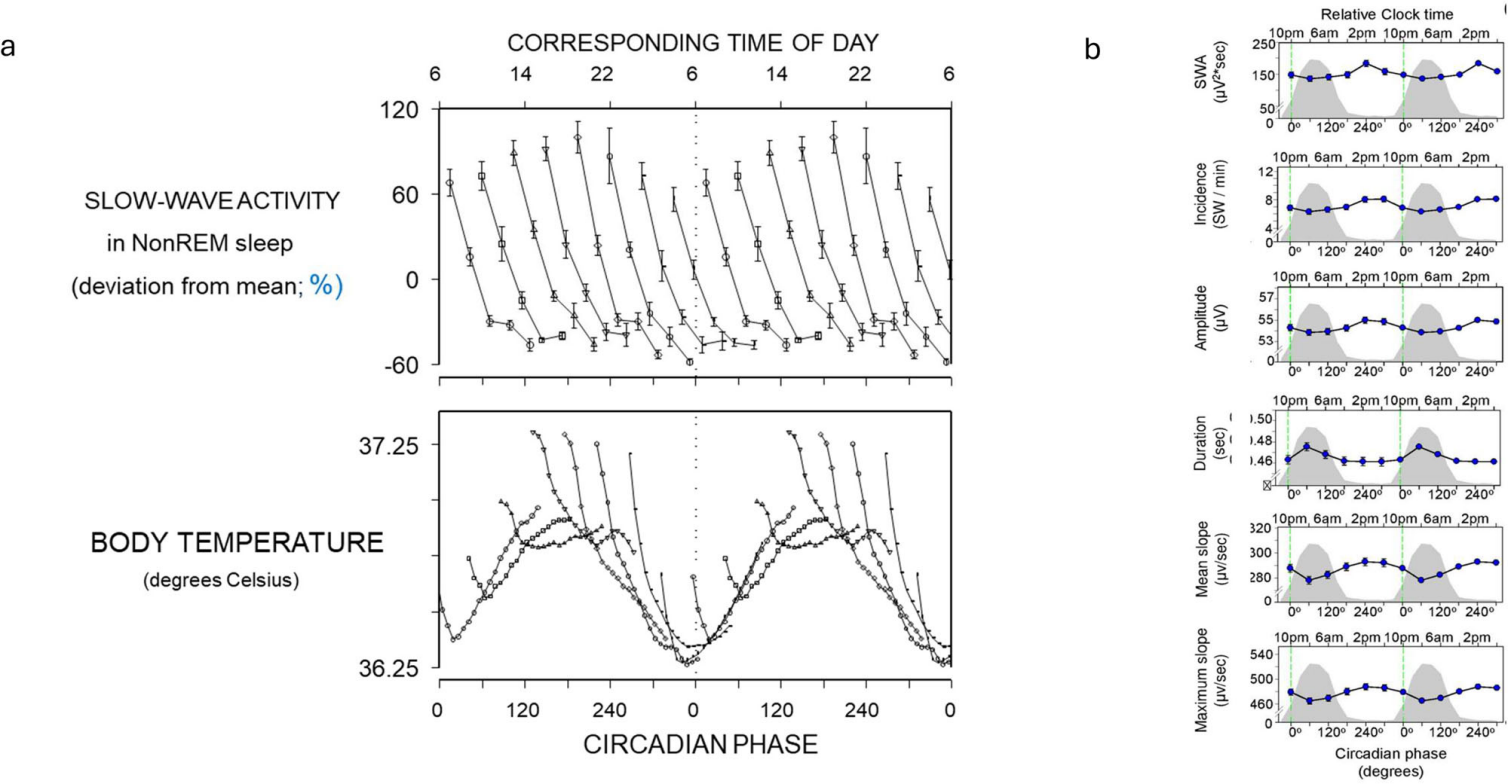
Dynamics of SWA during sleep are initiated across the circadian cycle. **a** Time course of SWA and core body temperature during a sleep episode initiated at different circadian phases (Circadian Phase 0 is the minimum of the circadian fit to core body temperature data). Data were collected during forced desynchrony of the sleep-wake cycle and the circadian rhythms of core body temperature and melatonin, in which the period of the imposed sleep-wake cycle was 28 h. Upper panel: Each 9 h and 20 min sleep period was divided into 5 equal parts and SWA during NREM sleep was averaged for each of these parts. Note how SWA declines in all sleep episodes at all circadian phases, but the initial values are lower when sleep is initiated between approximately midnight and 8 am. Bottom panel: Core body temperature during sleep episodes initiated across the circadian cycle. The first data point is the value of core body temperature just before lights out. The data illustrate both the circadian modulation of core body temperature and the temperature-lowering effects of sleep. Modified from ref. ^63^. **b** Circadian modulation of SWA characteristics. EEGs recorded during sleep periods in a forced desynchrony protocol were analysed to characterise the characteristics of slow waves. Slow wave parameters computed are SWA (power), incidence, amplitude, duration, and mean and maximum slope. Data are plotted relative to circadian phase as derived from the melatonin rhythm. The melatonin profile is indicated in grey. Modified from ref. ^65^. Note circadian modulation of these slow wave parameters.

Experiments in Zurich^41^, Harvard^63,64^, and Surrey^65^, in which sleep was initiated in the morning after sleep deprivation or the sleep-wake cycle was desynchronised from circadian rhythmicity in forced desynchrony protocols, unequivocally established that SWA declines during sleep at all circadian phases (Fig. 6a). These findings are in strong support of SWA being a marker of the hourglass process, driven by the sleep-wake cycle rather than the circadian process and confirm this aspect of the 2PM. However, evidence for a circadian influence, albeit smaller than the sleep-dependent influence, on SWA and other aspects of the sleep EEG was reported in all these studies. In a detailed analysis of the characteristics of slow waves, it was shown that SWA, the incidence of slow waves, their amplitude, duration and mean and maximum slope were all modulated by circadian phase (Fig. 6) and that this modulation was especially pronounced over central and posterior brain regions. Surprisingly, the forced desynchrony experiments also demonstrated that daytime sleep is characterised by more SWA and steeper slopes of slow waves compared to nighttime sleep, even when controlling for the confound of the circadian variation in wake and sleep duration. Similar observations have been reported in nocturnal rodents. Apparently, the circadian process does not promote slow waves when sleep occurs in phase with the circadian rhythm, i.e. for humans during the biological night, but during this circadian phase rather suppresses slow waves and promotes sleep spindles and REM sleep^64^. From a functional perspective, we’d be inclined to assume that sleep regulatory processes in a diurnal species are designed to optimise nocturnal sleep. Within this context, the circadian suppression of slow wave and the enhancement of spindles and REM sleep when sleep occurs during the circadian night may suggest that sleep spindles and REM sleep are important for good quality sleep.

The circadian modulation of SWA, albeit small, is at odds with the independence of the sleep-wake dependent process as indexed by SWA and circadian processes, which is a key tenet of the 2PM.

Another set of observations that suggests that circadian processes affect the dynamics of the sleep-wake dependent process and vice versa stems from studies on the effects of clock-gene variants on sleep phenotypes and underlying processes. For example, a Variable Number Tandem Repeat (VNTR) variant in the clock gene PER3 affects SWA^66^, the cognitive decline during sleep deprivation and restriction^48^, and fMRI assessed brain responses to a performance task across a 40 h period of sleep deprivation^67^. Within the context of the 2PM, these data are most parsimoniously explained by assuming that this variant and circadian processes affect the time constant of the build-up of process S^68^. Similar observations on the inter-relations between clock genes and sleep homoeostasis have been made for rodents^10,69^. Thus, also at the genetic level, sleep and circadian rhythmicity are entangled.

## The circadian sleep-wake propensity rhythm

### Maximum circadian drive for wakefulness in the evening

**Fig. 7:**
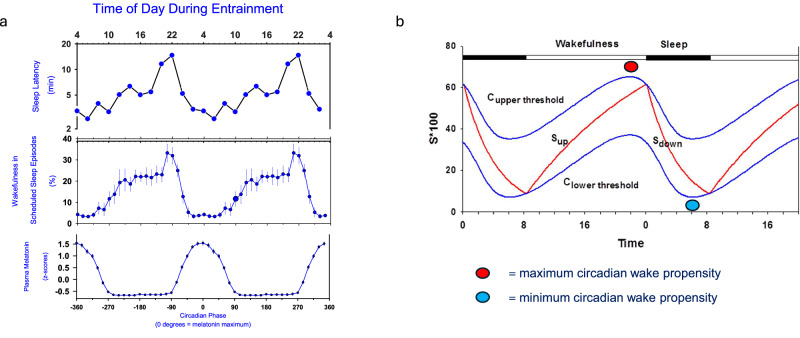
Circadian rhythm in sleep-wake propensity. **a** Sleep latencies and wake in scheduled sleep episodes plotted relative to the melatonin rhythm. Both the circadian drive to stay awake (i.e. long sleep latencies) and the circadian drive to wake up from sleep (i.e. wakefulness during scheduled time in bed periods) reach a maximum just before the onset of melatonin secretion, i.e. in the late evening). Sleep latencies are plotted on a logarithmic scale. Modified from ref. ^77^. **b** Two-process model of sleep regulation with modified circadian thresholds, with maximum circadian drive for wakefulness at 22:00 h and minimum drive for wakefulness at 06:00 h. With these thresholds, Process S hits the upper threshold just after the maximum drive of wakefulness when sleep is initiated and just after the minimum of the circadian threshold, when sleep is terminated (Dijk, previously unpublished figure).

Does the circadian process wake you up, put you to sleep or keep you awake and asleep? The circadian sleep propensity process, i.e. process C, was represented by a simple sine wave in 2PM-1982. In fact, in 2PM-1982, the negative function of C, i.e. the circadian wake propensity function, was plotted with a maximum circadian drive for wakefulness at 16:00 h. In 2PM-1984 process C (plotted as a wake propensity rhythm), was represented as a skewed sine wave with a maximum of the circadian wake drive at 14:00 h and a minimum at 05:00 h (See Fig. 1a). The skewness was introduced to account for the circadian variation of experimentally displaced sleep^70^. Thus, in both versions of the original 2PM, the maximum circadian drive for wakefulness (minimum drive for sleep) was assumed to be in the afternoon. How well does this fit the empirical observations? It certainly seems at odds with the notion of a mid-afternoon dip in alertness and the short sleep latencies when naps are taken in the afternoon during the Multiple Sleep Latency Test. But in these studies, time awake and time of day change simultaneously, and sleep latencies can therefore not be taken as an indicator of circadian sleep propensity. Early observations in shorter than 24 h sleep-wake cycle protocols and spontaneous desynchrony studies in which sleep-wake cycles could be much longer than 24 h suggested that the maximum circadian drive for wakefulness was located in the evening, rather than the morning or afternoon^71–73^. This phase of a strong circadian drive for wakefulness was referred to as the ‘forbidden zone for sleep’ or ‘the wake maintenance zone (WMZ)’. Having been exposed to the 2PM and its circadian thresholds for several years, and after our observations that people could sleep through the forbidden zone for sleep in the evening, albeit after sleep deprivation^38^, I was sceptical about the forbidden zone for sleep and wake maintenance zone. In the quest to test the predictions of the 2PM, I collected and analysed sleep data in forced desynchrony experiments in Charles Czeisler’s lab. Forced desynchrony experiments, originally designed to assess the intrinsic period of the human circadian pacemaker, also presented an opportunity to truly separate sleep-wake dependent and circadian components and interpret them in the context of the 2PM. This is because in forced desynchrony experiments, nearly all combinations of S and C are realised^74^. The analyses of the sleep data unequivocally, but much to my surprise, demonstrated that the data did not fit the waveforms of the circadian process as proposed in both 2PM-1982 and 2PM-1984 papers. The data showed that the circadian drive for wakefulness, as measured by both the propensity to initiate and terminate sleep and in the absence of the confounding effects of ‘sleep homoeostasis’ on sleep propensity, is highest in the evening hours, at approximately 22:00 h, i.e. close to the rise of melatonin (Fig. 7a). The circadian drive for sleep as indexed by sleep latency and sleep continuity, was highest close to the temperature nadir (5–6 am), a few hours before habitual waketime time. This waveform of the circadian sleep-wake propensity rhythm is consistent across young adults, adolescents, older people, in men, women and in blind individuals^57,75–78^ although individual differences exist. Thus, the circadian process doesn’t wake us up in the mornings and doesn’t put us to sleep in the afternoon or at the beginning of the night. Rather, the circadian process keeps us awake and keeps us asleep. More formally, the functional interpretation of this paradoxical phase relationship between the sleep-wake cycle and the circadian sleep-wake propensity rhythm is that the circadian process counters the increase in sleep pressure during the day and counters the dissipation of sleep pressure at night, thereby generating a consolidated sleep-wake cycle. This conceptualisation is closely related to the ‘opposing processes’ view of sleep regulation as proposed by Edgar and colleagues^79^. This view was also in part represented in early and later papers by Borbely and colleagues. In particular, after becoming familiar with the results of the forced desynchrony studies, the shape of the upper but not the lower threshold was changed to accommodate the diurnal variation in sleep latency^80^. However, the circadian process as described in the 2PM1982 and 2PM 1984, in which the drive for wakefulness reaches a maximum in the afternoon, continues to be represented in many presentations and reviews^5^. A ‘2PM model’ with revised circadian thresholds is presented in Fig. 7b and similar waveforms of the circadian process are implemented in a model in which effects of light, circadian rhythmicity and sleep homoeostasis are integrated^23^.

### How the interaction between the circadian process and the sleep-wake dependent process consolidates sleep

**Fig. 8:**
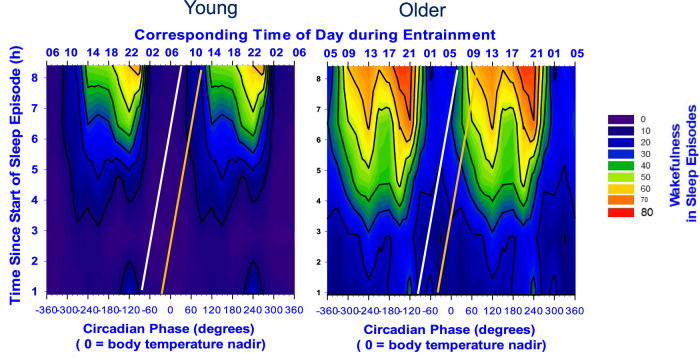
Interaction of time since start of sleep episode and circadian phase on wakefulness during the sleep opportunity period in young (left) and older (right) participants. Sleep disruption is always low at the beginning of sleep periods, and increases as sleep progresses, but much less so when sleep is initiated in the biological evening (white trajectory) compared to the biological day (orange trajectory). Older participants’ sleep is much more disrupted by misalignment of sleep and the circadian sleep-wake propensity rhythm than younger participants’ sleep. White trajectory: habitual sleep time. Orange trajectory: sleep delayed by approximately 4 h. (reanalysis of data in ref. ^77^).

Given the revised circadian waveform of sleep-wake propensity and given that sleep-wake dependent sleep propensity is highest at the beginning of sleep and then dissipates, consolidation of the sleep-wake cycle can only be achieved when the sleep-wake cycle is precisely timed with respect to the circadian cycle. How the interaction of the dissipation of sleep pressure and circadian rhythmicity shapes consolidation of sleep is illustrated in Fig. 8. Sleep is consolidated at the beginning of sleep episodes irrespective of the circadian phase at which it is initiated, because sleep pressure is high. However, only when the end of the time in bed period coincides with the maximum circadian sleep propensity, i.e. around the temperature minimum, will sleep remain consolidated throughout the time in bed period. Assuming an 8-h sleep period, this implies that for sleep to remain consolidated, it should be initiated just after the wake maintenance zone. When it is initiated in the early morning hours, as night shift workers often do, sleep becomes disrupted after only a few hours of sleep.

Comparing how the interaction between the circadian sleep-wake propensity rhythm and the sleep-dependent process affects sleep consolidation differs between young and older people yielded some interesting results. In healthy ageing—with an emphasis on healthy—the circadian influence on sleep consolidation becomes more prominent, such that in older, compared to young people, sleep is much more disrupted when sleep is not in precise alignment with circadian rhythmicity. This is most likely primarily related to a reduced wake-dependent increase in sleep pressure in older people^81^. An age-related reduction in the circadian promotion of sleep in the early morning may however also contribute to reduced sleep consolidation and early morning awakening in ageing. In this context, it should be pointed out that whether the circadian system primarily promotes wakefulness or also actively promotes sleep remains an area of contention. Analyses of the comparison of the circadian regulation of sleep in young and older people and analyses of sleep in rats indicates that the circadian system not only promotes wakefulness during the day but also promotes sleep and in particular REM sleep at night^77,82^.

### Physiological correlates of the circadian sleep-wake propensity rhythm

What are the physiological mechanisms generating this circadian rhythm of wake propensity? The circadian drive for wakefulness diminishes abruptly close to the onset of melatonin secretion and based on non-human primate studies close to the drop in orexinergic activity^77,83,84^. Human studies have demonstrated that administration of melatonin, melatonin agonists, and orexin antagonists can ‘silence’ the wake promoting signal^85–89^. Body and brain temperature should also be considered as correlates of the circadian sleep-wake propensity rhythm. In fact, there is a remarkable similarity in the diurnal waveform of human brain temperature and the circadian drive for wakefulness^90^.

Overall, the data confirm the 2PM concept that sleep propensity results from the interaction of a sleep-wake dependent process and a circadian process. The observed waveform of the circadian sleep-wake propensity rhythm is however at odds with the waveform as proposed in the 2PM-1982 and 2PM-1984.

## Circadian rhythmicity’s contribution to physiological, behavioural and molecular processes and homoeostasis

One frequently repeated interpretation of the 2PM is that ‘sleep is for homoeostasis and circadian rhythmicity for timing’. However, many physiological, cellular and molecular processes are not only affected by time awake or time asleep but also by circadian rhythmicity. This has been demonstrated by analysing the time course of a variety of variables during total sleep deprivation, partial sleep deprivation and forced desynchrony protocols.

### Direct effects of circadian rhythmicity

Circadian rhythmicity directly affects brain function. For example, fMRI assessment of brain responses to a cognitive task demonstrated that 49 brain areas (approximately 25 per hemisphere) display circadian rhythmicity^91^. This study in which in 33 healthy participants,13 functional magnetic resonance imaging sessions were scheduled across the circadian cycle, during 42 h of wakefulness and after recovery sleep, also identified 41 human brain areas which are responsive to time awake Other studies demonstrated that markers of EEG connectivity such as theta and alpha oscillations in the EEG during wakefulness not only increase with time awake but are also modulated by circadian phase^92^. In the evening hours (during the wake maintenance zone) connectivity is lower than predicted by the duration of prior wakefulness^93^. Excitability of the cortex as probed by Transcranial Magnetic Stimulation increases with time awake but is also markedly modulated by circadian phase, such that it is low in the wake maintenance zone^94^. Deterioration of brain function following repeated partial sleep deprivation and when time awake related sleep pressure is high, is substantially attenuated during the wake maintenance zone^48,95^.

Forced desynchrony studies revealed that many aspects of waking function, such as sleepiness, effort, sustained attention, accuracy, speed, executive function, EEG alpha oscillations, positive affect, are not only dependent on the duration of wakefulness but are also in part under direct circadian control^57,83,92,96,97^.

### Effects of circadian rhythmicity mediated by sleep

The results in the previous section demonstrate a direct circadian modulation of physiological variables, and brain function. Forced and spontaneous desynchrony studies have also shown circadian rhythmicity exert effects on sleep beyond sleep timing. Sleep characteristics modulated by circadian rhythmicity include SWA, REM sleep and sleep spindles. The relative magnitude of the circadian and sleep-wake dependent modulation varies across sleep parameters, with REM sleep and spindles under substantial circadian control^63^. These latter two aspects of sleep have repeatedly been shown to contribute to sleep functions such as memory consolidation. Thus, circadian rhythmicity through its effects on the macro and micro-structure of sleep contributes to processes essential to the recovery of brain function and the maintenance of homoeostasis. To what extent other aspects of the microstructure of sleep, such as, for example, the coupling of the slow oscillation and sleep spindles, functional connectivity, or EEG criticality measures, are modulated by circadian processes has not been established.

## Interactions of circadian rhythmicity and sleep-wake dependent processes shape overt rhythmicity

### Many non-sleep variables are also modulated by sleep-wake dependent and circadian processes

The 2PM was focussed on the regulation of sleep timing and in essence the two processes interacted only two times per day: i.e. at the transitions from wakefulness to sleep and the transition from sleep to wakefulness. Extended wakefulness and sleep affect many non-sleep variables. Likewise, circadian rhythmicity also modulates a multitude of behavioural, physiological and molecular variables. How sleep-wake dependent and circadian processes interact continuously in the regulation of all these variables, with the exception of sleepiness, was not addressed in the original version of the 2PM. One of the many positive impacts of the 2PM has been that protocols were designed to assess the separate contribution of the sleep-wake dependent and circadian process on a multitude of variables. Constant routine and forced desynchrony protocols are prime examples of protocols used to address this question. A conclusion from these human experiments is that nearly all variables are influenced by both the sleep-wake dependent and circadian processes. The relative contribution of the two processes, however, varies across all the non-sleep variables. In essence, the data show that overt rhythmicity is shaped by the continuous interaction of the two processes. Thus, at any point in time, the value of any variable is influenced by both sleep-wake history and circadian phase.

### The direct ‘masking’ effects vs sleep-wake history effects

In most interpretations of the 2PM the sleep-wake dependent effects relate to time awake and time asleep, i.e. sleep-wake history or sleep-homoeostasis. However, sleep and wake also have direct effects on physiology. These effects were not explicitly addressed in the 2PM but are represented by the term ‘masking’ in Fig. 1a of the 2PM-1984. These direct effects, however, should be considered within the concept that overt rhythmicity is shaped by the interaction of processes driven by the sleep-wake cycle and processes driven by circadian rhythmicity. Core body temperature may serve as an example. When the sleep-wake cycle is in phase with the temperature cycle, i.e. sleep occurs at night, the amplitude of the core body temperature rhythm is high. When sleep is absent, as during sleep deprivation, the amplitude of the rhythm is lower, but it is even lower when sleep is out of phase, i.e. occurs during the day (See Fig. 6a)^74^. Such direct effects have been observed for many variables, including hormones such as growth hormone, heart rate, etc. However, it is often difficult to separate the sleep-wake history effects of the direct ‘masking effects’, i.e. recovery effects of sleep may be related to the brain temperature-lowering effect of sleep, and the detrimental effects of prolonged wakefulness may be related to brain temperature being elevated during sleep deprivation.

### What happens when the sleep-wake cycle and circadian rhythmicity are misaligned?

As described above, separation of the two-process has shown that overt rhythmicity is shaped by both circadian and sleep-wake dependent components. This implies that alteration in the timing of these two processes relative to each other alters the observed rhythm.

### Interaction of sleep-wake dependent and circadian processes and individual differences in susceptibility to misalignment

**Fig. 9:**
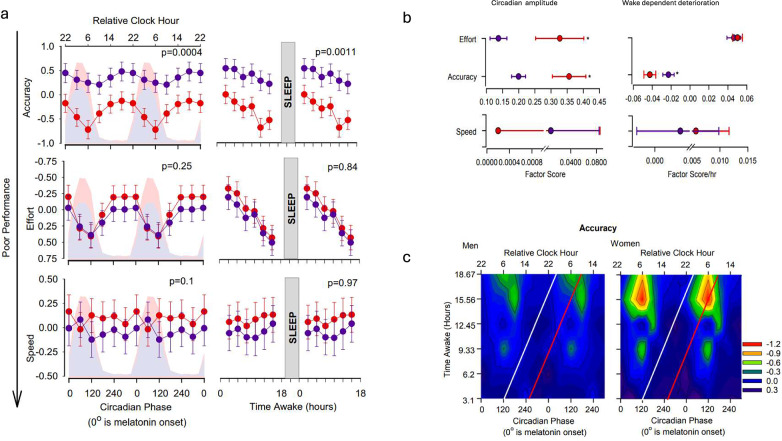
Sex differences in the circadian and sleep-wake dependent regulation of cognition. **a** Circadian and wake-dependent effects on principal components of cognition labelled Accuracy, Effort and Speed (blue: men, red: women). Melatonin profiles are represented as grey (men) and orange (women) area plots. Note the larger circadian amplitude of Effort and Accuracy in women compared to men. **b** Estimates of circadian amplitude and the slope of wake duration-dependent deterioration of performance in men and women. **c** Interaction between Time Awake and Circadian Phase on Accuray in men (left panel) and women (right panel). White Trajectory: ‘Day Shift’; Red Trajectory: ‘Night Shift’. Modified from ref. ^57^.

It has been repeatedly demonstrated that cognitive performance is impaired when assessed at night, that this impairment is exacerbated by sleep loss and that individuals differ in their susceptibility to these manipulations. Considering the interactions between circadian rhythmicity and the sleep-wake dependent process can contribute to the interpretation of the phenomena and individual differences. For example, differences in the circadian and sleep-wake dependent modulation of waking function have been described in a comparison of men and women. When women perform cognitive tasks during the biological night, their accuracy is more impacted than the accuracy of performance in men (Fig. 9a–c). Quantitative analyses of the interaction between circadian rhythmicity and the sleep-wake dependent process indicate that for cognitive domains such as effort, accuracy and speed, the differences between men and women are primarily related to a stronger circadian signal in women compared to men (Fig. 9a, b)^57^.

### Circadian and sleep-wake dependent processes don’t add up

**Fig. 10:**
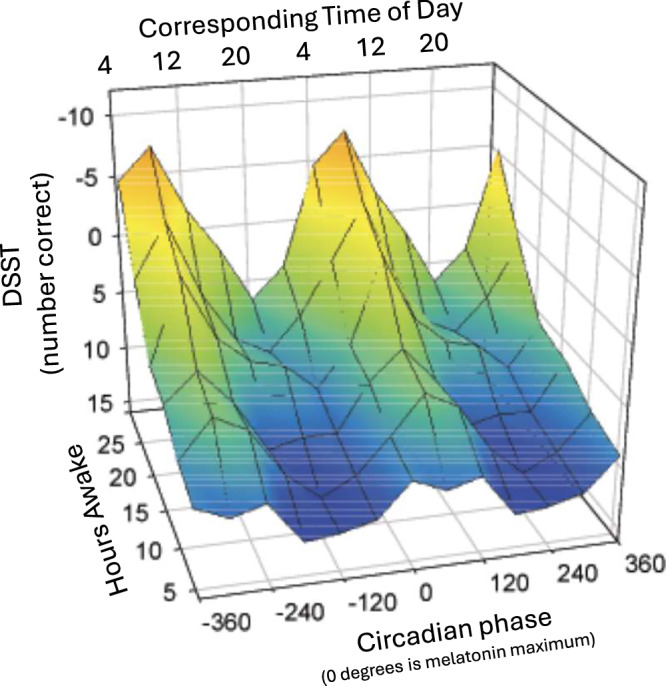
Interaction between time awake and circadian phase on waking performance as measured by the digit symbol substitution task. Performance (note reversed axis, up = poor performance) was assessed in a forced desynchrony protocol with a 42.85 h sleep-wake cycle. Note that a small circadian amplitude was observed when performance was assessed after only a few hours of time awake and an increase in amplitude with increasing time awake, with prominent deterioration of performance when assessed after the melatonin maximum and maintenance of performance in the evening hours. Modified from ref. ^100^.

In the original versions of the 2PM, the circadian and sleep-wake dependent processes were added up or subtracted to predict variables of interest, such as, for example, sleepiness. Forced desynchrony experiments demonstrated that the propensity to wake up from sleep, as indexed by the percentage of time awake while in bed, increases with time asleep, but the rate of this increase varies with circadian phase. This implies that the propensity to wake up is not a simple linear function of the sum of S and C. This non-additive nature of the interaction of S and C was first described for alertness assessed by a visual analogue scale and a 2-digit addition calculation performance test^97^. The non-additive interaction interpretation of this phenomenon was challenged and discussed early on^98,99^. The phenomenon has since been observed for many variables, and in many experimental paradigms^48,57,100,101^. In a forced desynchrony study in which the sleep-wake cycle had a period of 42.85 with 28.57 h scheduled for wakefulness and 14.28-h for sleep, effects of having been awake for 25 h has only small effects on performance on the Digit Symbol Substitution Task when it is performed at 22:00 h, i.e. in the wake maintenance zone. Brain function is also relatively preserved at 6:00 if the brain has been awake for only 5 to 10 h. On the contrary, the brain is very much impaired at 06:00 after 20–25 h of wakefulness (Fig. 10). The interaction between the 604 effects of ‘acute’ time awake, i.e. the duration of the current wake episode, and circadian phase is 605 amplified by chronic sleep loss^101^.

The effects of sleep loss and circadian phase furthermore interact with the performance task used to probe brain function. As shown by many investigators, simple aspects of brain function, such as alertness/sleepiness, are much more sensitive to sleep loss and the interaction with circadian phase misalignment than more complex functions such as working memory (Fig. 4e)^48^. In fact, repeated brain imaging sessions during sleep deprivation across the circadian cycle revealed that whereas the bold signal was altered in more than 40 brain areas when participants engaged in a simple sustained attention task, this was reduced to just one brain area when, under the same conditions, participants engaged in a complex working memory task^91^. These findings have practical implications for the optimal scheduling of shiftwork and the development of countermeasures. Recognising these interactions provides a theoretical framework for interventions such as taking a nap before the night shift. The nap-associated reduction in sleep pressure indeed leads to better simulated night shift performance^102^.

### Circadian and sleep-wake dependent effects on the transcriptome

Considering the separate contribution of the sleep-wake cycle and circadian rhythmicity has also led to new insights into the regulation of molecular processes in the brain and in the periphery. The human blood transcriptome shows aspects related to duration of wakefulness and circadian control. Under constant routine condition, in the absence of sleep, 7–9% of the blood transcriptome displays 24-h rhythmicity^103^. Acute sleep deprivation changes the levels of transcripts of as many as 856 genes in human blood.

**Fig. 11:**
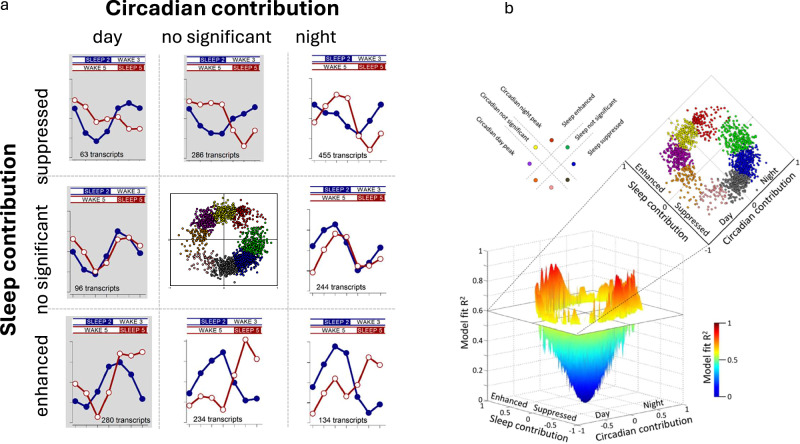
How sleep and circadian rhythmicity influence the blood transcriptome. **a** Illustrative time course of transcripts when sleep occurs at night (blue) or during the day (red). Transcripts can be suppressed, enhanced or not be affected by sleep. Transcripts may be driven by the circadian pacemaker to peak during the day, night or are not significantly affected. The observed time course is a result of the combined influence of sleep and circadian rhythmicity. For example, a transcript that is suppressed by sleep but is driven by the circadian system to peak at night, will have a high amplitude rhythm when sleep occurs during the biological night and a near-zero amplitude when sleep occurs during the day (top left panel in **a**). **b** Goodness of fit (R2) of a model which combines a direct sleep-wake influence and a circadian influence on timeseries of approximately 40 K transcripts in blood. from ref. ^111^.

In fact, the peripheral transcriptome can be used to estimate the phase of the central circadian pacemaker and to some extent the duration of time awake^104,105^. Direct effects of sleep are, however, also very prominent. Thus, when sleep occurs at night, and the amplitude of the temperature rhythm is high, approximately 6.4% of the human peripheral transcriptome is rhythmic. This includes transcripts of genes, the transcription of which is sensitive to temperature. When sleep is desynchronised from the sleep-wake cycle, the temporal organisation of the transcriptome is markedly disrupted, such that only 1% of the transcriptome is rhythmic. This reduction may in part be driven by the reduction in the amplitude of the temperature rhythm^106^. The contribution of the sleep-wake cycle and circadian rhythmicity to overt rhythmicity in the transcriptome can be quantified. Sleep may either acutely suppress, enhance or have no effect on the transcript abundance. Likewise, circadian rhythmicity may drive transcripts to peak during the day or night or have no effect (Fig. 11a). It shows that the extent to which rhythmicity is driven by the sleep-wake cycle, circadian rhythmicity or their interaction varies across transcripts(Fig. 11a, b). Some of the putative mechanisms underlying this regulation have been described, and quantitative models for this circadian and sleep-wake dependent regulation have been developed^106,107^. Overall, these data demonstrate the value of applying the core concept of the 2PM, i.e. separating the contribution of the sleep-wake cycle from the circadian contribution, for the understanding of altered rhythmicity in peripheral rhythms and the potential consequences of these alterations for health.

The interaction between the effects of chronic and acute sleep loss on waking performance^101^ is also present in the response of the peripheral transcriptome to acute sleep deprivation. Whereas in well-rested participants, the transcripts of only 122 genes were affected, 856 genes were affected when participants had been on a 6 h time in bed schedule for one week prior to the total sleep deprivation. Many of the genes affected by sleep deprivation were related to circadian rhythms, sleep homoeostasis, oxidative stress and metabolism^103^.

Recognition of these not-so-simple interactions between chronic sleep loss, acute sleep loss, sleep timing and circadian rhythmicity may help to understand the mechanisms underlying negative health outcomes related to shift work and insufficient sleep.

## Concluding remarks

The impact of the 2 PM on sleep and circadian research adds up to an impressive variety of experiments and rich data sets in a variety of species, relevant to many areas of basic neuroscience and human health. Maybe the most important contribution of the 2PM is that it inspired experiments to separately consider and manipulate the sleep-wake cycle and circadian rhythmicity, and this is especially so in humans. This has shown that processes driven by the sleep-wake cycle and circadian rhythmicity together shape rhythmicity in behaviour and physiology.

The naming of these processes and attribution of separate functional significance to them can be considered confusing. The notion of ‘sleep homoeostasis’ drew attention to aspects of the regulation of sleep independent of its circadian regulation. The current use of the phrase sleep homoeostasis seems at odds with the role behaviours play in biological control systems. Behaviours are mechanisms by which to maintain homoeostasis. Drinking is to maintain appropriate osmolarity and fluid volume, not a homoeostatic endpoint in itself, and drinking homoeostasis is not a meaningful term; see for example^6^. The notion that, within the context of sleep regulation and beyond, circadian rhythmicity is primarily or only for timing seems at odds with the wide variety of processes and variables, including aspects of the micro and macro-structure of sleep, driven by circadian oscillators.

**Fig. 12:**
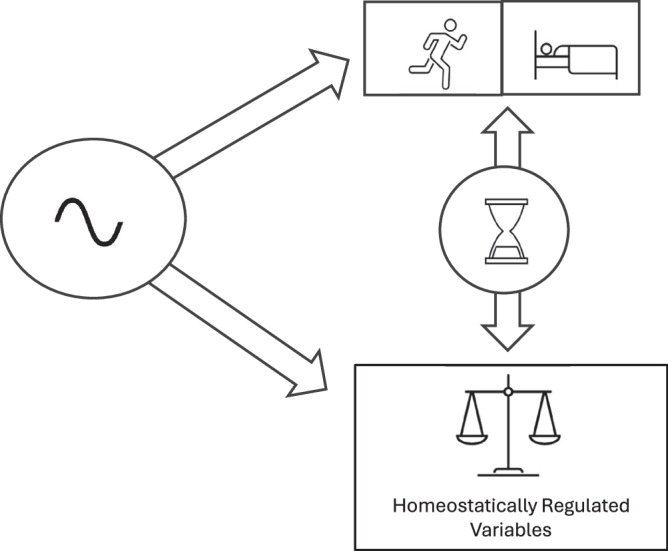
Schematic representation of how circadian rhythmicity and sleep contribute to homoeostasis. Sleep timing and sleep structure modulate many molecular and physiological processes and thereby contribute to homoeostasis. The circadian process contributes to homoeostasis by direct sleep-independent effects on many molecular and physiological processes. Other effects of circadian rhythmicity on homoeostasis are mediated by circadian effects on sleep timing and sleep structure.

Maybe we should instead consider that many variables under homeostatic control are influenced by the sleep-wake cycle and the circadian system. The sleep-wake cycle is regulated by an hourglass oscillator and input from the circadian pacemaker. Circadian processes impact homeostatically regulated variables either directly or through circadian effects on these variables, which are mediated through circadian influences on the timing and structure of sleep. The functional interpretation is that such a system accomplishes both optimal timing of behaviour, i.e. occurrence of consolidated bouts of behaviour at the appropriate time of day and drives physiological and molecular processes to support the behaviours and thereby contributes to homoeostasis of the brain and body. In more general terms, this can be depicted as a cartoon in which the ultimate goal is homoeostasis which is accomplished by circadian rhythmicity through direct pathways or through sleep, the propensity and structure of which is regulated by both circadian rhythmicity and an hourglass process (Fig. 12).

In a very early version of the 2PM, Alexander Borbely himself invoked a devil’s advocate (Advocatus Diaboli) to raise an anticipated critique of the 2PM. The Advocatus Diaboli said ‘While simplified diagrams of such a complex phenomenon as sleep may have an appealing quality, they may be seriously misleading or even outright wrong’^108^. Now, 40 years later, we can say that although the devil’s advocate was wrong, it is worthwhile revisiting some of their arguments. Overall, the 2 PM continues to have an appealing quality; the circadian rhythm vs. hourglass process remains fruitful but we also need to endorse the notion that ‘models should be simple but not too simple’ and not let the use of jargon such as ‘sleep homoeostasis or ‘circadian rhythmicity’ stand in the way of an understanding of the complexity of sleep-wake regulation of brain function and physiology. It may also be advisable not to restrict the search for correlates of ‘sleep homoeostasis’ to one aspect of sleep, such as SWA, but consider other aspects, such as REM sleep as well. Indeed, the Advocatus Diaboli invoked by Alexander Borbely stated, ‘…, the arguments advanced for the hypothesis show a striking neglect of REM-sleep. There is a marked REMS-homoeostasis.’

There is a continuing need to identify the mechanisms by which circadian rhythmicity influences sleep timing, structure, homeostatic endpoints, and the loci of interactions between S and C. Whereas in the 1980s sleep-inducing substances and their effects on SWA and sleep timing were much ‘en vogue’, over the decades sleep research has become more and more focused on neural networks, connectivity and synapses. Novel approaches searching for EEG measures beyond SWA that may reflect the recovery process taking place during sleep or the computational capacity of networks during wakefulness hold promise. Analysing the result from these approaches within the framework of sleep regulation by a sleep-dependent and circadian process may be useful to further our understanding of how sleep and circadian rhythmicity interact to serve brain function in humans.

Until then, we won’t know how and why we fall asleep and wake up, at least for now.

## Data Availability

No datasets were generated or analysed during the current study.
